# Adolescent cocaine self-administration induces habit behavior in adulthood: sex differences and structural consequences

**DOI:** 10.1038/tp.2016.150

**Published:** 2016-08-30

**Authors:** L M DePoy, A G Allen, S L Gourley

**Affiliations:** 1 Department of Pediatrics, Emory School of Medicine, Emory University, Atlanta, GA, USA; 2 Yerkes National Primate Research Center, Emory University, Atlanta, GA, USA; 3 Graduate Program in Neuroscience, Emory University, Atlanta, GA, USA; 4 Department of Psychiatry and Behavioral Sciences, Emory School of Medicine, Emory University, Atlanta, GA, USA

## Abstract

Adolescent cocaine use increases the likelihood of drug abuse and addiction in adulthood, and etiological factors may include a cocaine-induced bias towards so-called ‘reward-seeking' habits. To determine whether adolescent cocaine exposure indeed impacts decision-making strategies in adulthood, we trained adolescent mice to orally self-administer cocaine. In adulthood, males with a history of escalating self-administration developed a bias towards habit-based behaviors. In contrast, escalating females did not develop habit biases; rather, low response rates were associated with later behavioral inflexibility, independent of cocaine dose. We focused the rest of our report on understanding how individual differences in young-adolescent females predicted long-term behavioral outcomes. Low, ‘stable' cocaine-reinforced response rates during adolescence were associated with cocaine-conditioned object preference and enlarged dendritic spine head size in the medial (prelimbic) prefrontal cortex in adulthood. Meanwhile, cocaine resilience was associated with enlarged spine heads in deep-layer orbitofrontal cortex. Re-exposure to the cocaine-associated context in adulthood energized responding in ‘stable responders', which could then be reduced by the GABA_B_ agonist baclofen and the putative tyrosine receptor kinase B (trkB) agonist, 7,8-dihydroxyflavone. Together, our findings highlight resilience to cocaine-induced habits in females relative to males when intake escalates. However, failures in instrumental conditioning in adolescent females may precipitate reward-seeking behaviors in adulthood, particularly in the context of cocaine exposure.

## Introduction

Cocaine addiction is characterized by maladaptive decision-making and a loss of control over drug consumption; these changes may reflect the combination of preexisting behavioral characteristics and the effects of repeated drug exposure on prefrontal cortical neurobiology that then further exacerbate disease psychopathology.^1, 2^ Adolescents are particularly vulnerable to drugs of abuse—for example, adolescence is characterized by both high rates of experimental drug use and heightened susceptibility to the development of dependence.^3, 4^ Adolescent-emergent drug use is also associated with increased likelihood of abuse and dependence in adulthood, as well as decreased likelihood of seeking treatment.^5, 6^ Thus, a better understanding of the effects of adolescent cocaine exposure on prefrontal cortical-dependent decision-making is critical.

In both humans and rodents, adolescents and adults appear to be differentially sensitive to cocaine,^7^ but investigations into the long-term consequences of adolescent drug exposure are lacking. Here we isolated individual differences in low-dose cocaine self-administration patterns in adolescent mice and then characterized neurobehavioral outcomes in adulthood. We focused on dendritic spines within the prefrontal cortex, so-called ‘reward seeking' following abstinence, and the development of habit-like response strategies, considered an etiological factor in the maintenance of drug seeking in cocaine-abusing individuals.^2^

Throughout, we tested male and female mice because sex appears to contribute to both drug sensitivity and the progression of drug taking to drug dependence. For example, although more men use cocaine, more women develop dependence.^8^ In therapeutic settings, women report greater craving, consumption of larger quantities and higher lifetime abuse rates than men.^9^ Sex is also a determinant in drug-related decision-making—for example, habitual alcohol seeking develops more rapidly in chromosomal males than chromosomal females.^10^

Here, we developed an oral cocaine self-administration technique for adolescent mice. Although orally ingested cocaine has lower bioavailability than injected cocaine, it nonetheless readily penetrates the brain,^11^ and oral cocaine self-administration has been used previously in rats and nonhuman primates.^12, 13, 14, 15^ Moreover, an advantage of this approach over intravenous delivery methods is that it allows for a non-drug control group that robustly responds for reinforcement, and here, it allowed us to feasibly screen large populations of mice and isolate individual differences in cocaine self-administration. We discovered that a history of cocaine self-administration induced habit-like behavioral inflexibility and preference for a cocaine-associated object in adulthood, but only in certain populations. Moreover, these effects were sex-dependent. Although the relatively low doses of cocaine ingested did not impact dendritic spine densities in deep-layer prefrontal cortex, dendritic spine head size—a predictor of synapse formation—was modified, with larger orbitofrontal cortical spine heads in habit-resilient mice and larger prelimbic cortical spine heads in mice prone to habit-based behaviors. Last, we identified strategies to prevent context-induced ‘reward seeking' following abstinence, focusing on candidate drugs identified in prior reports—the GABA_B_ agonist, baclofen and the putative trkB agonist, 7,8-dihydroxyflavone (7,8-DHF).

## Materials and methods

### Subjects

A total of 262 C57BL/6 mice (Jackson Labs, Bar Harbor, ME, USA) or transgenic mice expressing *thy1*-derived yellow fluorescent protein and bred onto a C57BL/6 background were used.^16^ The mice were maintained on a 12-h light cycle (0800 h on), experimentally naive, and provided food and water *ad libitum* between testing phases. The procedures were Emory University IACUC-approved. Exact *n*'s for every experiment are provided in Supplementary Figure 1.

### Cocaine self-administration

The mice self-administered cocaine using illuminated Med Associates (Georgia, VT, USA) conditioning chambers with two nose-poke recesses and a retractable lever. The mice were ‘pre-trained' to lever-press during two daily 1 h sessions in which the lever was extended for the duration, and one press resulted in 10% w/v sucrose (100 μl). Next, the mice were transitioned to a chained response requirement such that nose poking on the active recess resulted in extension of the lever for 10 s (fixed ratio 1). Upon lever-press, 10% w/v sucrose with 0, 7.5 or 75 μg ml^−1^ cocaine was delivered via automated dipper that otherwise rested in a trough containing the remaining solution. Reinforcer delivery coincided with extinction of a light within the active aperture. Sessions ended at 60 min or when the mice acquired 30 reinforcers. The volume of the remaining liquid in the trough decreased daily.

Sucrose-reinforced lever-press training was initiated on P28–29 (for ‘adolescents') or P40 (for ‘periadolescents' Supplementary Figure 2).^17^ Cocaine was introduced at P30–31 or P42, respectively. Feeding was titrated such that the mice maintained 100% of their expected body weights according to C57BL/6 growth curves (Jackson Labs). The mice were tested daily for 12 days, then mice were fed freely and undisturbed for 2 weeks.

### Response-outcome contingency degradation

As adults, mice were food-restricted to ~90% of their original body weight and trained to nose poke for food reinforcers in contextually distinct chambers. These chambers were equipped with two nose-poke recesses and a separate food magazine. Responding was reinforced using a fixed ratio 1 schedule wherein 30 pellets were available for responding on each of two nose-poke recesses (60 pellets per session). The mice acquired the responses within five to eight 70 min sessions (one per day), indicated graphically. Response acquisition curves represent both nose-poke responses per minute.

Response-outcome contingency degradation was accomplished similarly to our prior reports.^18, 19, 20, 21, 22^ In the 25 min ‘non-degraded' session, one nose-poke aperture was occluded, and responding on the other aperture was reinforced using a fixed ratio 1 schedule. In the 25 min ‘degraded' session, the opposite aperture was occluded, and reinforcers were delivered into the magazine at a rate matched to each animal's reinforcement rate the previous day. Responses produced no programmed consequences, thus ‘degrading' the response-outcome contingency. These sessions, and the location of the ‘degraded' aperture within the chamber, were counter-balanced. This two-day process was repeated, and then, both apertures were available during a 10 min probe test conducted in extinction. The response rates during this test are shown, as is the common practice. Preferential responding on the ‘non-degraded' aperture is indicative of goal-directed action selection, whereas a failure to differentiate between responses that are more, vs less, likely to be reinforced is considered habitual.^23^

### Matched cocaine injections

We injected a group of behaviorally naive female mice intraperitoneally daily from P31–42 with doses of cocaine that were matched to either ‘stable responders' or ‘escalating responders' in the prior 7.5 μg ml^−1^ cocaine self-administration cohort (1 ml per 100 g). The mice were left undisturbed until testing 2 weeks later.

### Context-induced cocaine seeking

The mice with a history of adolescent cocaine self-administration (7.5 μg ml^−1^) were placed in the chambers in which they had originally self-administered cocaine. The light within the active aperture was lit, and the reinforcer-associated lever was extended for the duration of the 30 min session. Lever pressing resulted in the dipper that previously delivered liquid, however, no reinforcer was available.

In experiments aimed at pharmacologically reducing these behaviors, the sessions were shortened to 15 min to accommodate repeated testing. The mice were pretreated with vehicle or baclofen (1.25 mg kg^−1^, in water, intraperitoneally, 1 ml per 100 g) 30 min before test. Using a randomized design, the mice were re-tested 2 days later with vehicle or 7,8-DHF pretreatment (5 mg kg^−1^, in 17% dimethyl sulfoxide, intraperitoneally, 1 ml per 100 g) 60 min before test.

### Cocaine-conditioned object preference

Adolescent female mice acquired 7.5 μg ml^−1^ cocaine reinforcers as described. Then, cocaine-conditioned object preference was tested by injecting mice with either cocaine (10 mg kg^−1^, intraperitoneally) or saline and placing the mice immediately following the injection into locomotor monitoring chambers (Med Associates) containing a cocaine- or saline-paired object, as appropriate. The objects were a wrapped 1 ml syringe barrel and 15 ml Falcon tube.

The mice were injected with both cocaine and saline daily for 5 days, with test sessions separated by 3 h. The order of these injections and which item was paired with cocaine was randomized. The following day, the mice were placed in the chambers drug-free for 10 min. Each object was adhered to the floor at either end of the chamber. Time spent in the cocaine–object quadrant relative to the saline–object quadrant and in nasal or oral contact with the objects was quantified.

### Dendritic spine imaging and enumeration

Adolescent female yellow fluorescent protein-expressing mice acquired 7.5 μg ml^−1^ cocaine reinforcers as described. After instrumental contingency degradation testing, the mice were killed by rapid decapitation. Yellow fluorescent protein-expressing brains were submerged in chilled 4% paraformaldehyde for 48 h, then transferred to 30% w/v sucrose, followed by sectioning into 50 μm-thick sections on a microtome held at −15 °C. Unobstructed dendritic segments were imaged on a spinning disk confocal (VisiTech International, Sunderland, UK) on a Leica microscope. Z-stacks were collected with a × 100 1.4-NA objective using a 0.1 μm step size, sampling above and below the dendrite. After imaging, we confirmed at × 10 that the image was collected from the intended region.

Collapsed z-stacks were analyzed using ImageJ: Each protrusion <4 μm was considered a spine.^24^ Bifurcated spines were considered singular units. To generate density values, spine number for each segment was normalized to the length of the segment. Dendritic spine head diameters were generated by measuring the distance across the widest part of the spine head on collapsed z-stack images. Only clearly discernible heads were measured. Spines were considered mushroom-like or thin-type based on head diameter above or below 0.5 μm (adapted from refs 24, 25). In control mice, proportions of thin-type vs mushroom-like spines were in line with those previously reported from mice and nonhuman primates, using both *ex vivo* and tissue culture approaches.^25, 26, 27, 28^

Six to 10 independent segments from secondary branches within 50–150 μm of the soma were collected. Each animal contributed a single density value (its average) to statistical analyses, and spine head metrics were treated as populations. In females, dendritic spine head sample sizes were 482 sucrose, 378 stable and 553 escalating responders in the prelimbic cortex and 589 sucrose, 541 stable and 634 escalating responders in the orbitofrontal prefrontal cortex (oPFC). In male mice, the sample sizes were 243 sucrose, 244 stable and 281 escalating responders (also oPFC).

Apical prelimbic cortical branches and basilar oPFC branches (layer V) were imaged because Kolb and colleagues indicate that psychostimulants regulate dendritic spine density in these regions.^29, 30, 31^ In addition, dendritic spine density in these regions is associated with stressor-related habits and related behavioral phenotypes.^22, 32^ A single blinded rater imaged and scored spines.

### Statistical analyses

**Figure 1:**
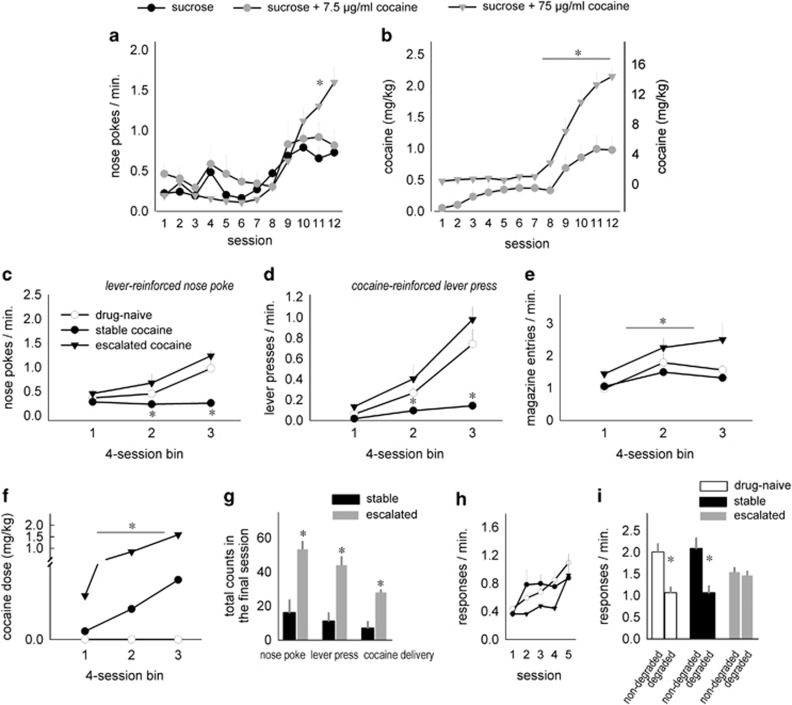
Adolescent mice will respond for cocaine (p.o.), inducing habit-based behavior in adulthood. (**a**) Adolescent mice respond for access to a lever that, when pressed, results in a liquid sucrose reinforcer. The addition of 7.5 μg ml^−1^ cocaine does not impact response rates, and 75 μg ml^−1^ cocaine increases response rates. (**b**) Daily cocaine doses are shown. Note that the right *y* axis in gray refers to the 75 μg ml^−1^ cocaine group. (**c**) Male adolescent mice respond on a nose-poke aperture to gain access to a lever that, when pressed, results in the delivery of a liquid reinforcer containing sucrose ±7.5 μg ml^−1^ cocaine. A median split of total nose pokes in the cocaine group highlights that approximately half of the mice develop very low, stable response rates, whereas the other half develop response rates that increase across adolescence. (**d**) These mice similarly escalate in responding on the cocaine-reinforced lever. (**e**) Magazine head entries are also highest in the cocaine-escalating group. Notably, despite low nose-poke response rates, the ‘stable responders' enter the magazine as frequently as sucrose-reinforced mice. (**f**) Cocaine doses are shown. (**g**) The final day of testing in cocaine-reinforced mice is summarized; the mice classified as ‘escalating' respond more on the nose-poke aperture, more on the cocaine-reinforced lever and acquire more cocaine deliveries. (**h**) In adulthood, the same mice are trained in distinct chambers to nose poke on two discrete nose-poke apertures for food reinforcers. Response acquisition curves represent both responses per minute. (**i**) Sucrose-reinforced and stable mice are subsequently sensitive to modifications in the predictive relationship between a response and its outcome, as indicated by preferentially engaging the response most likely to be reinforced following instrumental contingency degradation (‘non-degraded'). Mice with a history of escalating cocaine self-administration, however, fail to modify response selection strategies, generating both responses equally in a habit-based manner. Means+s.e.m., *P<0.05.

In all the experiments except our initial dose-response curve reported in Figure 1, cocaine-reinforced mice were split into ‘stable responders' and ‘escalating responders' based on a median split of total nose pokes. Throughout, response rates and dendritic spine densities were compared by analysis of variance with repeated measures when appropriate. Following the interactions or main effects between greater then two groups, Tukey's *post hoc* comparisons were applied and are indicated graphically. Adjustments for multiple comparisons were not required. Comparisons of cocaine doses did not include non-drug control groups (0 mg kg^−1^ per day) to avoid an artificial reduction in variance. Total cocaine-reinforced responses in males were compared by two-tailed *t*-tests. In experiments assessing context-induced cocaine ‘seeking', response rates were normalized to those generated on the last day of cocaine self-administration. When this normalization resulted in non-real numbers (twice), data points were excluded. Throughout, data points +2 s.d. from the mean were also excluded, with this exclusion criterion pre-established. *P*<0.05 was considered significant; *P*-values <0.1 but >0.05 are noted as trends. Dendritic spine head diameters were compared by Kolmogorov–Smirnov (K–S) tests; because of the high degree of power generated in these analyses, only *P*<0.001 was considered significant. Means+s.e.m. or individual data points are represented in the figures.

Sample sizes were determined on the basis of power analyses and pilot studies using independent animals; these pilot experiments provided initial findings that were then replicated at least once in the course of these experiments. Additional replications can be found within the manuscript itself. The only exceptions were dendritic spine studies, in which hundreds of samples were included, and conditioned-object preference studies, in which case, object preference was confirmed across multiple sessions in the same animals (session 1 is shown).

## Results

### Mice orally self-administer cocaine

We developed an oral cocaine self-administration protocol that eliminates the need for surgical placement of an intravenous catheter in very small adolescent mice. In addition, unlike intravenous self-administration, oral self-administration methods offer a comparison group that actively responds for a non-drug control solution. To initially validate our method, we trained adolescent (female) mice to perform a nose-poke→lever-press chain to acquire a 10% w/v sucrose reinforcer with or without cocaine. Response acquisition curves indicate that, as in rats and nonhuman primates, adolescent mice respond for orally delivered cocaine (12–15; Figure 1a). Moreover, responding is dose-sensitive, with mice nose poking at higher rates for 75 μg ml^−1^ cocaine relative to 0 and 7.5 μg ml^−1^ (interaction F_(22,517)_=2.95, *P*<0.001; Figure 1a). As expected, the daily doses of cocaine acquired were also higher in mice responding for the higher concentration (interaction F_(11,308)_=46.6, *P*<0.001; Figure 1b). In the interest of clarity, response rates and dosing will be presented in four-session bins hereafter, and a table of experiments and exact *n* values is shown in Supplementary Figure 1.

Next, we trained male adolescent mice to respond for 7.5 μg ml^−1^ cocaine reinforcers. We segregated cocaine-reinforced mice on the basis of a median split of total nose-poke responses, highlighting two populations of mice: those that generated response rates that escalated across adolescence, and those that generated moderate, largely stable response rates that resembled cocaine avoidance (interaction for nose-poke: F_(2,20)_=6, *P*=0.009; for lever-press: F_(2,20)_=4.9, *P*=0.02; Figures 1c and d). These mice will be referred to as ‘escalating' and ‘stable' throughout.

Escalating mice entered the magazine more frequently than other groups (main effect F_(2,20)_=7, *P*<0.05; Figure 1e) and acquired higher doses of cocaine throughout (main effect F_(1,12)_=8.5, *P*=0.01; Figure 1f). Total cocaine-reinforced responses from the final session are also shown, highlighting higher response counts in escalating mice (all *P*-values <0.05; Figure 1g).

Prior reports indicate that a history of cocaine exposure in older male rodents causes stimulus–response habits and habit-based behavioral inflexibility,^12, 19, 33, 34, 35^ but the long-term effects of adolescent cocaine self-administration are largely untested. The mice were thus left undisturbed for 2 weeks, then trained as adults to respond on two operandi for food reinforcers in contextually distinct testing chambers. All the mice acquired the new responses; response acquisition curves represent both responses per minute (Figure 1h). We identified a trend for session × group interaction (F_(2,20)_=2.8, *P*=0.08), but no significant differences. We then modified the predictive relationship between one response and the associated food reinforcer such that responding was no longer likely to be reinforced (response-outcome contingency degradation). Both sucrose-reinforced and stable cocaine mice readily differentiated between responses that were more vs less likely to be reinforced and preferentially engaged the highly reinforced response in a goal-directed manner (Figure 1i). By contrast, mice with a history of escalating cocaine self-administration were insensitive, instead responding habitually on both apertures (interaction F_(2,20)_=4.5, *P*<0.05; Figure 1i). Response rates between this group and sucrose-reinforced mice were very similar during adolescence, suggesting that developmental cocaine, and not response history, induces inflexible habits in adulthood.

### In females, stable, not escalating, cocaine self-administration induces habit biases

**Figure 2:**
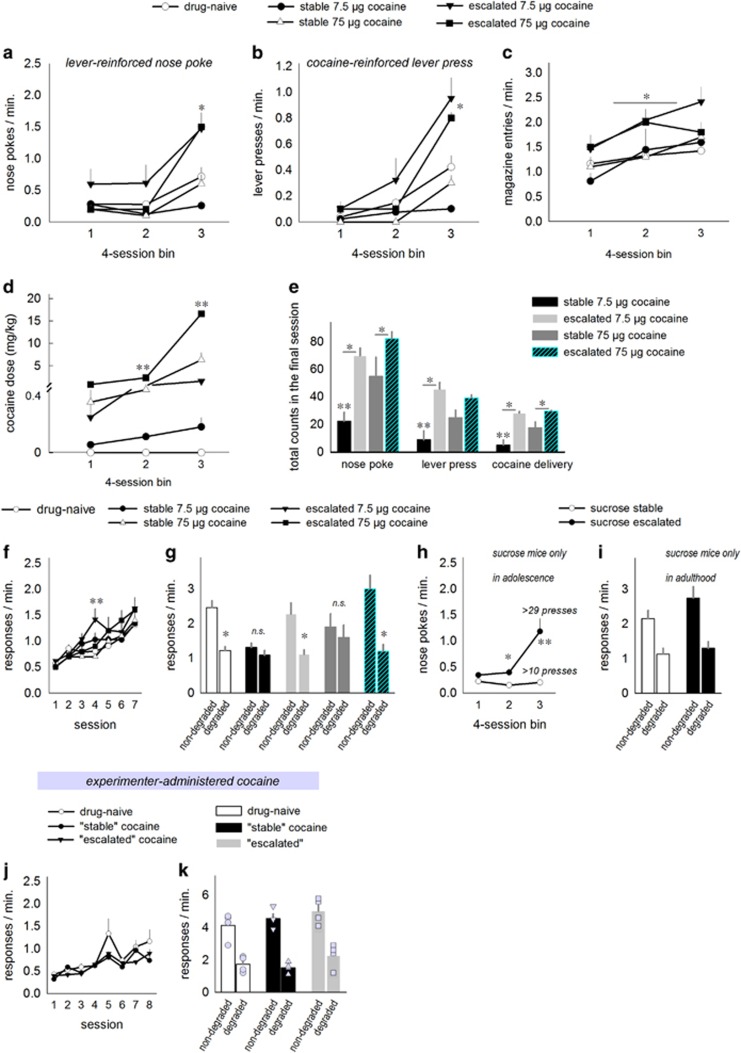
Adolescent cocaine self-administration patterns, not dosing, determine decision-making strategies in female mice. (**a**) Female mice respond for a liquid reinforcer containing sucrose ±7.5 or 75 μg ml^−1^ cocaine. A median split of total nose pokes (by concentration) highlights that approximately half of the mice develop very low, stable response rates, whereas the other half develop an escalating response pattern. (**b**) These mice similarly escalate in responding on the cocaine-reinforced lever. (**c**) Magazine head entries are also highest in the cocaine-escalating groups. Notably, despite low nose-poke response rates, the ‘stable responders' enter the magazine with equivalent frequency relative to sucrose-reinforced mice. (**d**) Accumulated cocaine doses per session are also shown. Note that throughout, the ‘escalating' responders lever-pressing for 7.5 μg ml^−1^ cocaine acquire the same dose of cocaine as the ‘stable' mice acquiring 75 μg ml^−1^ cocaine, thus the key difference between these groups is the pattern of responding, rather than the dose of cocaine acquired. (**e**) The final day of training is also summarized in cocaine-reinforced mice; the mice classified as ‘escalating' respond more on the nose-poke aperture, more on the cocaine-reinforced lever and acquire more cocaine deliveries than their respective ‘stable' counterparts. All the groups respond more than the 7.5 μg ml^−1^ ‘stable' group. (**f**) In adulthood, the same mice respond on two distinct nose-poke apertures for food reinforcers in separate chambers. Response acquisition curves represent both responses per minute. (**g**) Sucrose-reinforced and escalating mice are subsequently sensitive to modifications in the predictive relationship between a response and its outcome, preferentially engaging the response most likely to be reinforced following instrumental contingency degradation. Mice with a history of stable responding, regardless of cocaine dose, instead generate both responses equally, habitually. (**h**) The sucrose control group was then retroactively split, highlighting different nose-poke patterns across adolescence, ultimately culminating in ‘stable' mice generating roughly 10 sucrose-reinforced lever presses during the final four sessions and ‘escalating' mice generating ⩾29 responses. (**i**) Despite these individual differences, all sucrose mice were sensitive to instrumental contingency degradation. (**j**) Separate adolescent mice were injected with cocaine at doses matched to either the ‘stable' or ‘escalating' 7.5 μg ml^−1^ cocaine mice. In adulthood, these mice were trained to perform two distinct responses for food reinforcement, with no differences between groups. (**k**) Next, the likelihood of one of the responses being reinforced was decreased. Unlike with cocaine self-administration, all groups preferentially generated the response more likely to be reinforced in a goal-directed manner. Owing to small sample sizes, individual data points are represented, in addition to group means. Means+s.e.m., *P<0.05 vs control, **P<0.05 vs all the other groups.

We next conducted the same experiment in female mice, and we added groups of mice responding for a higher, 75 μg ml^−1^ cocaine concentration. Again, we divided each population of cocaine-reinforced mice on the basis of a median split of the total nose-poke responses; half developed an escalating response profile and half developed response rates that were stable by comparison (interaction F_(4,48)_=5.8, *P*=0.001; Figure 2a). Lever pressing followed the same pattern (interaction F_(4,48)_=8.6, *P*<0.001; Figure 2b), and as in males, escalating mice entered the magazine more than stable or control, sucrose-reinforced mice (main effect F_(4,48)_=3.04, *P*=0.03; Figure 2c) and accumulated larger doses of cocaine (interaction F_(3,28)_=74, *P*<0.001; Figure 2d). Also as in male mice, mice that generated stable, ‘low' response rates for the 7.5 μg ml^−1^ cocaine concentration responded less than sucrose-reinforced mice, a profile that could be considered cocaine avoidant.

Notably, the escalating 7.5 μg ml^−1^ group acquired the same daily doses of cocaine as the stable 75 μg ml^−1^ group (Figure 2d); the difference between these groups was thus largely in response pattern, and not dose ingested.

Figure 2e summarizes cocaine-reinforced response counts and reinforcement counts on the final day of testing. All the groups generated higher response and reinforcement counts than the stable low-dose 7.5 μg ml^−1^ group (interactions for nose-poke F_(3,26)_=18.9, *P*<0.001; lever-press F_(3,26)_=12.5, *P*<0.001; cocaine delivery F_(3,26)_=20.8, *P*<0.001).

In adulthood, all the mice acquired novel instrumental responses for food reinforcement. An interaction indicated that the escalating 7.5 μg ml^−1^ group generated response rates that were briefly elevated, but otherwise, there were no differences between the groups (F_(24,288)_=2, *P*=0.006; Figure 2f). By contrast, response selection strategies varied markedly: in this case, a history of stable responding, regardless of cocaine concentration, resulted in habit-based behavioral inflexibility. Meanwhile, female mice that developed escalating response profiles, again regardless of cocaine concentration, inhibited non-reinforced responses in a goal-directed manner (interaction F_(4,48)_=2.9, *P*=0.03; Figure 2g).

We next applied the same median split approach to the sucrose-consuming mice, revealing individual differences in nose poking during response acquisition in adolescence (interaction F_(2,38)_=8.6, *P*=0.001), corresponding to an average of 10 or 29 reinforced lever presses per session during the final four test sessions (Figure 2h). In adulthood, however, both low- and high-responding groups were behaviorally sensitive to instrumental contingency degradation (main effect of response F_(1,19)_=25.9, *P*<0.001; Figure 2i). Thus, the pattern of operant responding during adolescence influenced habit biases in adulthood in cocaine, but not sucrose, self-administering mice.

### Experimenter-administered low-dose cocaine does not induce habits

We next tested the effects of experimenter-administered cocaine exposure using a separate group of female mice. Cocaine was injected daily during adolescence, with doses and timing matched to the average daily dose that the 7.5 μg ml^−1^ stable responders or escalating responders acquired. As adults, these mice acquired food-reinforced responses with no differences between groups (Fs<1; Figure 2j). In addition, unlike with self-administered cocaine, all the mice were sensitive to modifications in response-outcome associative contingencies (main effect of response F_(1,9)_=28, *P*<0.001; Figure 2k).

### Response patterns during adolescence are associated with stimulus-elicited ‘reward seeking' and dendritic spine morphologies in adulthood

**Figure 3:**
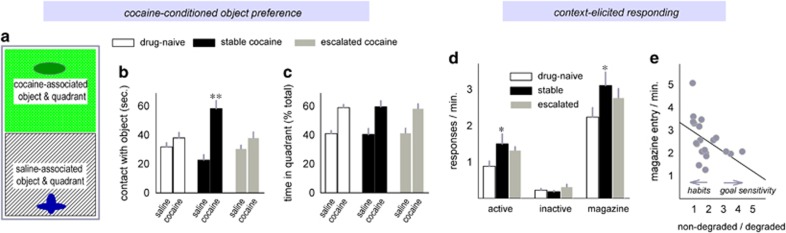
Behavioral response patterns in adolescence are associated with cocaine-conditioned object preference and context-elicited responding in adulthood. (**a**) Adult female mice with a history of adolescent cocaine self-administration were injected daily with saline and cocaine, and each injection was paired with a unique object. Subsequently, the mice were placed in a chamber with saline- and cocaine-associated objects adhered to either end. (**b**) Mice with a history of ‘stable' responding (and that developed habits) spent more time in physical contact with the cocaine-associated object. (**c**) All the mice spent more time in the quadrant containing the cocaine-associated object. (**d**) A separate cohort of cocaine-exposed female mice was tested for sensitivity to context-induced reinstatement-like behavior. Stable responders generated higher response rates than sucrose-reinforced control mice. Magazine head entries were also elevated, whereas responding on the inactive nose-poke aperture did not differ between groups. (**e**) Magazine head entries co-varied with decision-making strategies, in that the mice that preferentially generated the non-degraded response following instrumental contingency degradation (in a goal-directed manner) had fewer magazine head entries. Meanwhile, the mice that failed to differentiate between the degraded and non-degraded response contingencies—habitually—generated more magazine head entries. Means+s.e.m., *P<0.05, **P<0.001.

We next generated another group of cocaine self-administering female mice, focusing on the 7.5 μg ml^−1^ concentration that produced mice with low, stable response rates that could be considered cocaine avoidant (see again, Figure 2). We then paired injections of saline and cocaine with distinct objects. Cocaine-elicited locomotor activity progressively increased over days, evidence of locomotor sensitization (F_(4,48)_=13.05, *P*<0.001), and locomotor activity did not differ between groups (main effect and interaction Fs<1; Supplementary Figure 3). However, when we placed mice in the locomotor chambers with access to both objects during a drug-free probe test (Figure 3a), mice with a history of stable responding spent more, not less, time in physical (nasal and oral) contact with the cocaine-associated object (interaction F_(2,12)_=9.8, *P*=0.003; Figure 3b). All the mice spent more time in the quadrant containing the cocaine-associated object than the saline-associated object (F_(1,12)_=25.7, *P*<0.001; Figure 3c), confirming that the cocaine-conditioned object preference procedure was successful.

A separate group of mice was tested for sensitivity to context-elicited responding. As adults, mice were returned to the chambers in which they had been trained to acquire cocaine. Again, stable responders generated higher, not lower, response rates on the previously active nose-poke aperture, whereas escalating responders did not differ from sucrose control mice (main effect F_(2,17)_=4.3, *P*=0.03; Figure 3d). Responding on the inactive nose-poke aperture did not differ between groups (main effect F_(2,17)_=1.5, *P*=0.3; Figure 3d), but magazine head entries were also elevated in the stable group (F_(2,17)_=8.5, *P*=0.003; Figure 3d), countering the perspective that this group's behavior during adolescence was a durable predictor of cocaine avoidance; rather, these behaviors (Figures 3b) are paradoxically suggestive of heightened cocaine seeking.

Finally, head entry rates co-varied with behavioral sensitivity to contingency degradation (*r*=0.45, *P*<0.05; Figure 3e). Specifically, mice that generated goal-directed response strategies had fewer magazine head entries than the mice that behaved in a habitual manner.

Adolescence corresponds with a period of considerable structural refinement in the PFC;^36, 37, 38, 39, 40^ also see ref. 4. In animal models, the absence of proteins involved in these processes can increase sensitivity to cocaine,^41^ and cocaine itself, at higher doses than those achieved here, is a potent regulator of dendritic spines. We thus imaged dendritic spines on deep-layer excitatory pyramidal neurons in the prelimbic and oPFC subregions in adult female mice that had acquired 7.5 μg ml^−1^ cocaine or sucrose throughout adolescence.

**Figure 4:**
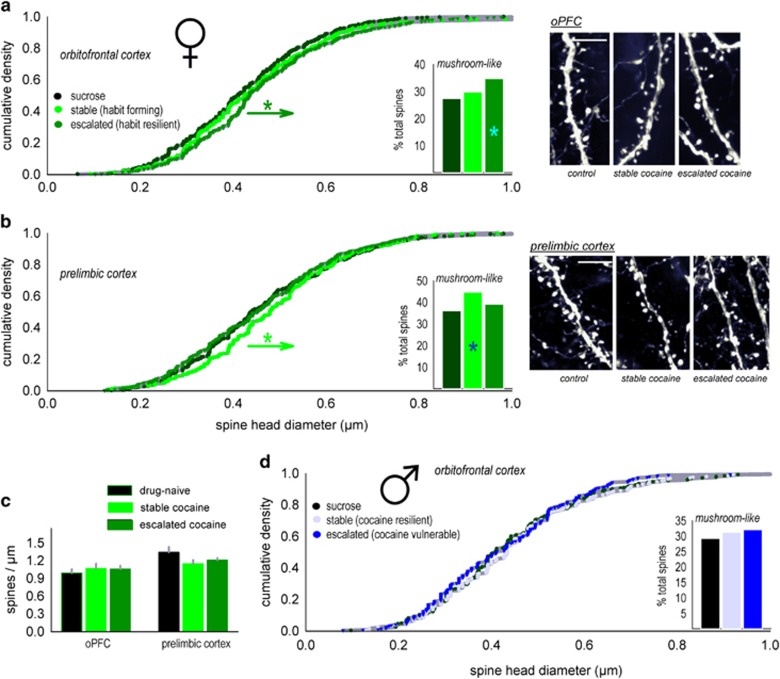
Habit-based vs goal-directed behavior: neuronal structural correlates. (**a**) Deep-layer neurons in adult female mice with a history of oral cocaine self-administration (7.5 μg ml^−1^) were imaged. Mice that developed goal-directed response strategies in adulthood—despite cocaine exposure—had larger dendritic spine heads in the oPFC. (**b**) By contrast, ‘stable' mice with inflexible, habitual response strategies had enlarged spines heads in the prelimbic cortex. (**c**) At the doses tested, there were no changes in dendritic spine densities. (**d**) Also, at the doses tested, cocaine did not impact oPFC dendritic spine head diameter in males. Representative dendrites adjacent. Scale bar, 5 μm. Bars represent means+s.e.m. *P<0.001 vs other groups. oPFC, orbitofrontal prefrontal cortex.

In the oPFC, dendritic spine head diameters were larger in habit-resilient, escalating mice (K–S, *P*<0.001; Figure 4a). By contrast, spine heads in habit-prone ‘stable' mice did not differ from control values (K–S, *P*=0.19). In the prelimbic cortex, a different pattern emerged: here, spine head diameters were larger in habit-prone ‘stable' mice relative to mice that did not develop habits (K–S, *P*<0.001; Figure 4b). As an additional measure, the spines were categorized into mushroom-like or thin-type spines. As would be expected, on the basis of the cumulative density analyses, habit resilience was associated with more mushroom-like spines in the oPFC, whereas in the prelimbic cortex, habit vulnerability was associated with more mushroom-like spines (Figures 4a and b, inset). Interestingly, at the low doses tested, there were no changes in overall dendritic spine densities (oPFC, F<1; prelimbic, F_(2,21)_=1.3, *P*=0.3; Figure 4c).

Together, this pattern suggests that spine head enlargement in the prelimbic cortex is associated with habit-based behaviors. (An alternative perspective is that cocaine impacted spine head diameters; in this case, however, it is unclear why a very low dose, but not a higher dose, would remodel prelimbic cortical spine heads.) Perhaps more provocatively, spine head enlargement in the oPFC is associated with habit resilience. To assess whether this effect was sex-selective, we also imaged dendritic spines in the oPFC of male mice. In this case, dendritic spine head diameters did not differ (K–S *P*=0.5 control vs escalating, and *P*=0.6 control vs stable; Figure 4d).

### Blockade of reward-seeking behavior

**Figure 5:**
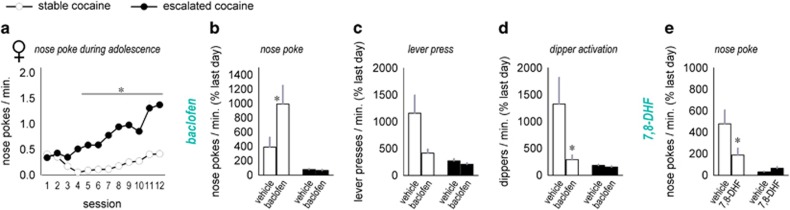
GABA- and trkB-targeted intervention strategies have differential effects in mitigating context-elicited reward-seeking behavior. (**a**) Adolescent female mice respond for a sucrose+cocaine (7.5 μg ml^−1^) reinforcer. A median split of total nose pokes highlights that approximately half of the mice develop low, stable response rates, whereas the other half develop an escalating response pattern. (**b**) The mice were then reintroduced to the conditioning chambers in adulthood. Mice with a history of stable responding generated robust response rates, relative to the final day of training. Baclofen further increased nose poking. (**c**) Responding on the ‘cocaine-taking lever' followed a different trend, however, and (**d**) activation of the dipper that previously delivered cocaine was significantly reduced. (**e**) By contrast, the trkB agonist 7,8-DHF reduced nose poking. Lever pressing and dipper delivery followed the same general trend, although interactions were nonsignificant (not shown). Means+s.e.m., *P<0.05.

We next attempted to reduce reward-seeking behaviors. Motivated by prior reports in the field (see the 'Discussion' section), we pretreated adult female mice that had self-administered sucrose+cocaine during adolescence (Figure 5a) with the GABA_B_ agonist baclofen or the trkB agonist 7,8-DHF. Baclofen did not reduce responding on the lever-associated nose poke aperture; in fact, responding increased in the stable group (interaction F_(1,32)_=4.4, *P*=0.04; Figure 5b). However, baclofen induced a trend towards reduced response rates on the cocaine-associated lever (interaction F_(1,30)_=3.7, *P*=0.06; Figure 5c), and this was accompanied by a significant reduction in activation of the dipper that had previously delivered reinforcement (interaction F_(1,30)_=4.4, *P*=0.04; Figure 5d). Throughout, the mice that had previously escalated in their responding generated low response rates as expected, and these already low rates were not impacted by baclofen.

The trkB agonist 7,8-DHF also mitigated reward-related responding, but in a different way: In this case, nose poking was reduced, specifically in the stable group (interaction F_(1,32)_=5.2, *P*=0.03; Figure 5e). Lever pressing and dipper presentation rates followed the same general pattern, although interaction effects did not reach significance (interaction lever-pressing F_(1,30)_=1.6, *P*=0.21; delivery rate F<1; not shown). Nonetheless, this pattern suggests that trkB stimulation reduces context-elicited reward-seeking behaviors.

### Are the behavioral effects of cocaine self-administration age-specific?

Last, we trained a group of older female periadolescent (P42; ref. 17) mice to self-administer 7.5 μg ml^−1^ cocaine. Notably, responding for cocaine was more homogenous, with no mice responding less than those acquiring sucrose, and additionally, no groups developed habit behaviors when tested later in adulthood. See Supplementary Figure 2.

## Discussion

Here we trained adolescent mice to respond for a sucrose solution containing cocaine. Some mice responded robustly, whereas others developed low ‘stable' response patterns that at times resembled cocaine avoidance. These individual differences served as a platform from which to isolate the influence of individual differences in cocaine self-administration patterns during adolescence on reward-related decision-making in adulthood. In male mice, escalated responding for cocaine was associated with an inability to select actions on the basis of their outcomes. Instead, these mice deferred to familiar habit-based response patterns. These findings build on evidence that acute, subchronic and prolonged cocaine exposure can result in habit-based behavior in older male rodents,^12, 19, 33, 34, 35, 42^ but they are the first to our knowledge to extend this work to the context of individual differences in cocaine self-administration in adolescence. Given that an estimated >90% of individuals with diagnosed substance use disorders began ingesting drugs of abuse in adolescence,^43^ this is a notable gap.

In female mice, very low rates of cocaine-reinforced responding were associated with habit behavior. To further validate this unexpected finding, we increased the concentration of cocaine 10-fold such that the stable responders were ingesting a substantially higher dose, on par with what the escalating responders had acquired in initial experiments. In addition, these new ‘stable' mice responded at higher rates and could not obviously be considered cocaine avoidant. Again, stable and not escalating responders developed inflexible habits in adulthood. In cocaine-dependent humans, self-reports suggest that cocaine self-administration is greatly motivated by drug-related stimuli in men, whereas women abuse cocaine to alleviate stress, that is, to fulfill a specific goal.^9^ Thus, escalating cocaine seeking may be linked to goal-directed decision-making biases in females. In support of this perspective, experimenter-administered cocaine, with dosing matched to mice self-administering cocaine, had no obvious effects, further suggesting that the pattern of responding, and not cocaine dosing, determines long-term outcomes. Even though sucrose-reinforced adolescent mice did not develop the same habit biases (Figures 2h and i), the influence of cocaine is still likely secondary to the influence of response patterns, given that cocaine dosing was quite low in several mice.

Next, we repeatedly paired experimenter-administered cocaine with a common laboratory object and saline with another object. When presented with both objects, ‘stable responders' spent more time in physical contact with the cocaine-associated object. When returned to the cocaine-associated chambers following a period of forced abstinence, these mice also entered the reinforcer-associated magazine with greater frequency than control and escalating counterparts. This pattern points to heightened sensitivity to cocaine-associated stimuli.

Taken together, it may be that the female mice that we refer to as ‘stable responders' are weak in associating a response and an outcome, their behaviors instead driven by stimulus–response associations. This could account for low response rates during adolescence and a reliance on stimulus–response habits in adulthood. Furthermore, when returned to the conditioning chambers in which mice had acquired reinforcers as adolescents, the context energized nose poking. Viewed through this lens, our findings could suggest that biases towards stimulus–response-driven behaviors in adolescent females precipitate reward-seeking behaviors in adulthood, particularly in the context of cocaine exposure.

In these experiments, we used a chained response schedule in which one response—nose poke—generated a lever that, when pressed, resulted in cocaine delivery. In adolescent mice, nose poking and lever-pressing were largely related, with more cocaine-taking associated with greater responding on the ‘cocaine-seeking' nose poke. In older, periadolescent mice, however, the cocaine-taking response did not differ between groups (Supplementary Figure 2). In other words, ‘stable' periadolescent cocaine self-administering mice were highly efficient, responding less for the cocaine-associated lever, but nonetheless acquiring as much cocaine as their escalating counterparts. These findings are parsimonious with evidence that cocaine self-administering periadolescent rats self-administer cocaine more efficiently than adults; this phenomenon may be related to pubertal neurobiology.^7^

### Structural predictors of decision-making strategies

Drugs of abuse remodel the structure of principal pyramidal neurons in the prefrontal cortex.^44^ For example, amphetamine and cocaine, at higher doses than acquired by our 7.5 μg ml^−1^ groups here, decrease dendritic spine density in the oPFC and cause proliferation in the dorsomedial prefrontal cortex.^29, 30, 31, 41, 45^ Further, adolescent cocaine exposure, followed by re-exposure in adulthood, enlarges the head diameter of remaining spines in the oPFC.^41^ We suggest that this is a neuroprotective response, given that female mice that maintained goal-orientated response strategies despite drug exposure had larger deep-layer oPFC dendritic spine heads here. Mice deficient in the cytoskeletal regulatory factor Abl2/Arg kinase fail to develop these larger spine heads following cocaine exposure, and notably, these mice could be considered cocaine-vulnerable—developing exaggerated locomotor sensitization and habit-like behavioral inflexibility in an instrumental reversal task.^41, 46^ Spine head size is intimately linked with synapse formation and stability;^47^ thus, resilience to repeated cocaine exposure may be associated with the elaboration or stabilization of oPFC synapses.

The finding that oPFC spines were unaffected in habit-prone mice is seemingly at odds with evidence that oPFC damage impairs response-outcome conditioning. For example, asymmetric lesions disconnecting the oPFC from the striatum impair animals' ability to make decisions on the basis of the predictive relationship between actions and their outcomes, as in ‘stable' mice here;^20^ see also ref. 48. Importantly, our findings do not rule out the contributions of factors other than deep-layer dendritic spines in the oPFC—such as layer II/III spines, transcription factors or other regulatory elements.^49^ In addition, adolescent cocaine exposure can reduce dendrite branching in the deep-layer oPFC,^50^ potentially reducing the overall number of spines and synapses. This would not be captured in density measures here, as spine count is normalized to dendritic segment length.

In males, oPFC dendritic spines were unchanged. By contrast, prior investigations using higher doses of cocaine revealed larger heads in cocaine-resilient males (as in resilient females here).^41^ Together, these data suggest that adolescent females may be more sensitive to drug-related structural modifications. Why might this be? Although dendritic spine densities may not differ by sex in the rodent cortex,^51, 52, 53^ during the early and pre-adolescent periods, dendritic spine proliferation and dendrite elaboration can be more pronounced in females than in males.^51, 52^ Subsequently, dendritic spine pruning is, in some cortical regions, also exaggerated in females relative to males, and this is not impacted by castration.^52, 53^ Higher rates of dendrite and dendritic spine stabilization and turnover in females may increase sensitivity to durable modifications in neuron structure. Higher turnover rates may also explain why, for example, nicotine exposure during adolescence modifies oPFC dendrite branching in females, but not males.^54^

Within the prelimbic cortex, dendritic spine heads were enlarged in ‘habit-vulnerable' mice that also developed reinstatement-like behavior. These deep-layer spines are innervated by the basolateral amygdala (BLA).^55^ Asymmetric inactivation of the BLA and prelimbic cortex (disconnecting these structures) and optogenetic inhibition of BLA inputs to the prelimbic cortex interfere with the reinstatement of cocaine self-administration.^56, 57^ Enlarged spine heads—and increased synaptic fidelity—in the prelimbic cortex could thus promote BLA-dependent reinstatement in vulnerable mice, as seen here. In addition, immediate-early gene activation in the medial prefrontal cortex–nucleus accumbens pathway is associated with cocaine vulnerability, defined by high rates of self-administration; inactivation of the prelimbic cortex corrects this vulnerability, consistent with considerable evidence that the prelimbic cortex energizes cocaine-reinforced behavior.^58^

As in the seminal studies that first reported that drugs of abuse remodel cortical dendritic spines,^59, 60, 61^ we did not monitor estrus cycle. We aimed to avoid untoward stressor-related confounds resulting from vaginal cell sample collection, particularly given that corticosteroids regulate the same dendritic spine populations of interest here.^22, 32, 62^ Also, dendritic spine tissue samples were collected from multiple independent groups, thus the likelihood that testing phases routinely coincided with the same estrus phase is low. Furthermore, sex differences in habit formation may be chromosomally—rather than gonadally—dependent. For example, chromosomal XX complement accelerates food-reinforced habits relative to XY complement,^63^ whereas chromosomal males rapidly develop alcohol-reinforced habits.^10^

### Blockade of context-elicited responding

Finally, we attempted to block reward-seeking behaviors. We were motivated by prior investigations: First, female rats selectively bred for high saccharin preference develop stable, rather than escalating, cocaine-reinforced response profiles in adolescence, similar to ‘stable' mice here.^64^ Nonetheless, these rats, as with our mice, can be more vulnerable to developing relapse-like behavior in adulthood.^65^ Baclofen interferes with reinstatement in even these vulnerable rats.^66^ Thus, we treated mice with baclofen, reducing presentation of the sucrose+cocaine-associated dipper, in concordance with prior reports.^66^ Our nose-poke→lever-press chain, however, revealed increased nose poking despite decreased lever-pressing. Why baclofen would have opposing influences on these behaviors in unclear, but it complicates the prediction that baclofen could be beneficial in clinical settings.

We also tested the trkB agonist 7,8-DHF. Although the roles that trkB and its high-affinity ligand brain-derived neurotrophic factor have in food and cocaine seeking are region-specific,^67, 68^ we were motivated by evidence that systemic 7,8-DHF treatment induces dendritic spine proliferation in the oPFC,^69^ and it enhances response-outcome conditioning (opposing habits)^69^ and the extinction of preference for a cocaine-associated context.^70^ Here, 7,8-DHF reduced context-elicited reward-seeking, warranting further investigations into its utility in braking reinstatement behaviors, particularly in animals with prior exposure to higher doses of cocaine that may more closely model doses ingested by cocaine-abusing humans.

Overall, we find that self-administration of cocaine at doses that escalate across adolescence is associated with a reliance on habit-based behavior in adulthood in males, but not females. Instead, low, stable rates of responding are associated with habit behavior in females. Focusing on mice generating the lowest response rates, we also found that these low response rates were likely not reflecting durable cocaine avoidance. Instead, low response rates, potentially stemming from failures in response-outcome conditioning, were associated with multiple behaviors interpreted as modeling aspects of problematic drug use—preference for cocaine-associated stimuli and reward-seeking behavior that was energized by reward-associated contextual cues. Sucrose-reinforced mice with ‘low' response rates did not develop the same behavioral patterns in adulthood, potentially pointing to complex interactions between preexisting behavioral traits and even very modest cocaine exposure in female adolescents.

## References

[bib1] Jentsch JD, Taylor JR. Impulsivity resulting from frontostriatal dysfunction in drug abuse: implications for the control of behavior by reward-related stimuli. Psychopharmacology 1999; 146: 373–390. 10550488 10.1007/pl00005483

[bib2] Everitt BJ. Neural and psychological mechanisms underlying compulsive drug seeking habits and drug memories—indications for novel treatments of addiction. Eur J Neurosci 2014; 40: 2163–2182. 24935353 10.1111/ejn.12644 PMC4145664

[bib3] Chambers RA, Taylor JR, Potenza MN. Developmental neurocircuitry of motivation in adolescence: a critical period of addiction vulnerability. Am J Psychiatry 2003; 160: 1041–1052. 12777258 10.1176/appi.ajp.160.6.1041 PMC2919168

[bib4] Anthony JC, Petronis KR. Early-onset drug use and risk of later drug problems. Drug Alcohol Depend 1995; 40: 9–15. 8746919 10.1016/0376-8716(95)01194-3

[bib5] Kessler R, Aguilar-Gaxiola S, Berglund P, Caraveo-Anduaga J, DeWit D, Greenfield S et al. Patterns and predictors of treatment seeking after onset of a substance use disorder. Arch Gen Psychiatry 2001; 58: 1065–1071. 11695954 10.1001/archpsyc.58.11.1065

[bib6] Kandel DB, Yamaguchi K, Chen K. Stages of progression in drug involvement from adolescence to adulthood: further evidence for the gateway theory. J Stud Alcohol 1992; 53: 447–457. 1405637 10.15288/jsa.1992.53.447

[bib7] Wong WC, Ford KA, Pagels NE, McCutcheon JE, Marinelli M. Adolescents are more vulnerable to cocaine addiction: behavioral and electrophysiological evidence. J Neurosci 2013; 33: 4913–4922. 23486962 10.1523/JNEUROSCI.1371-12.2013 PMC3630505

[bib8] O'Brien MS, Anthony JC. Risk of becoming cocaine dependent: epidemiological estimates for the United States, 2000-2001. Neuropsychopharmacology 2005; 30: 1006–1018. 15785780 10.1038/sj.npp.1300681

[bib9] Becker JB, Perry AN, Westenbroek C. Sex differences in the neural mechanisms mediating addiction: a new synthesis and hypothesis. Biol Sex Differ 2012; 3: 14. 22676718 10.1186/2042-6410-3-14 PMC3724495

[bib10] Barker JM, Torregrossa MM, Arnold AP, Taylor JR. Dissociation of genetic and hormonal influences on sex differences in alcoholism-related behaviors. J Neurosci 2010; 30: 9140–9144. 20610747 10.1523/JNEUROSCI.0548-10.2010 PMC2921163

[bib11] Pan WJ, Hedaya MA. An animal model for simultaneous pharmacokinetic/pharmacodynamic investigations: application to cocaine. J Pharmacol Toxicol Methods 1998; 39: 1–8. 9596142 10.1016/s1056-8719(97)00097-x

[bib12] Miles FJ, Everitt BJ, Dickinson A. Oral cocaine seeking by rats: action or habit? Behav Neurosci 2003; 117: 927–938. 14570543 10.1037/0735-7044.117.5.927

[bib13] Miles FJ, Everitt BJ, Dalley JW, Dickinson A. Conditioned activity and instrumental reinforcement following long-term oral consumption of cocaine by rats. Behav Neurosci 2004; 118: 1331–1339. 15598142 10.1037/0735-7044.118.6.1331

[bib14] Walker QD, Schramm-Sapyta NL, Caster JM, Waller ST, Brooks MP, Kuhn CM. Novelty-induced locomotion is positively associated with cocaine ingestion in adolescent rats; anxiety is correlated in adults. Pharmacol Biochem Behav 2009; 91: 398–408. 18790706 10.1016/j.pbb.2008.08.019 PMC2715835

[bib15] Macenski MJ, Meisch RA. Ratio size and cocaine concentration effects on oral cocaine-reinforced behavior. J Exp Anal Behav 1998; 70: 185–201. 9768506 10.1901/jeab.1998.70-185 PMC1284677

[bib16] Feng G, Mellor RH, Bernstein M, Keller-Peck C, Nguyen QT, Wallace M et al. Imaging neuronal subsets in transgenic mice expressing multiple spectral variants of GFP. Neuron 2000; 28: 41–51. 11086982 10.1016/s0896-6273(00)00084-2

[bib17] Spear LP. The adolescent brain and age-related behavioral manifestations. Neurosci Biobehav Rev 2000; 24: 417–463. 10817843 10.1016/s0149-7634(00)00014-2

[bib18] Hinton EA, Wheeler MG, Gourley SL. Early-life cocaine interferes with BDNF-mediated behavioral plasticity. Learn Mem 2014; 21: 253–257. 24737916 10.1101/lm.033290.113 PMC3994500

[bib19] Gourley SL, Olevska, Gordon J, Taylor JR. Cytoskeletal determinants of stimulus-response habits. J Neurosci 2013; 33: 11811–11816. 23864670 10.1523/JNEUROSCI.1034-13.2013 PMC3713723

[bib20] Gourley SL, Olevska A, Zimmermann KS, Ressler KJ, DiLeone RJ, Taylor JR. The orbitofrontal cortex regulates outcome-based decision-making via the lateral striatum. Eur J Neurosci 2013; 38: 2382–2388. 23651226 10.1111/ejn.12239 PMC3864662

[bib21] Gourley SL, Swanson AM, Jacobs AM, Howell JL, Mo M, Dileone RJ et al. Action control is mediated by prefrontal BDNF and glucocorticoids. Proc Natl Acad Sci USA 2012; 109: 20714–20719. 23185000 10.1073/pnas.1208342109 PMC3528547

[bib22] Swanson AM, Shapiro LP, Whyte AJ, Gourley SL. Glucocorticoid receptor regulation of action selection and prefrontal cortical dendritic spines. Commun Integr Biol 2013; 6: e26068. 24563705 10.4161/cib.26068 PMC3917952

[bib23] Balleine BW, O'Doherty JP. Human and rodent homologies in action control: corticostriatal determinants of goal-directed and habitual action. Neuropsychopharmacology 2010; 35: 48–69. 19776734 10.1038/npp.2009.131 PMC3055420

[bib24] Peters A, Kaiserman-Abramof IR. The small pyramidal neuron of the rat cerebral cortex. The perikaryon, dendrites and spines. Am J Anat 1970; 127: 321–355. 4985058 10.1002/aja.1001270402

[bib25] Young ME, Ohm DT, Dumitriu D, Rapp PR, Morrison JH. Differential effects of aging on the dendritic spines in visual cortex and prefrontal cortex of the rhesus monkey. Neuroscience 2014; 274C: 33–43. 10.1016/j.neuroscience.2014.05.008 PMC4108992 24853052

[bib26] Dickstein DL, Brautigam H, Stockton SD Jr., Schmeidler J, Hof PR. Changes in dendritic complexity and spine morphology in transgenic mice expressing human wild-type tau. Brain Struct Funct 2010; 214: 161–179. 20213269 10.1007/s00429-010-0245-1 PMC3032082

[bib27] Ren W, Liu Y, Li B. Stimulation of α_2A_-adrenoreceptors promotes the maturation of dendritic spines in cultured neurons of the medial prefrontal cortex. Mol Cell Neurosci 2012; 49: 205–216. 22015717 10.1016/j.mcn.2011.10.001

[bib28] Kroener S, Mulholland PJ, New NN, Gass JT, Becker HC, Chandler LJ. Chronic alcohol exposure alters behavioral and synaptic plasticity of the rodent prefrontal cortex. PLoS One 2012; 7: e37541. 22666364 10.1371/journal.pone.0037541 PMC3364267

[bib29] Robinson TE, Kolb B. Persistent structural modifications in nucleus accumbens and prefrontal cortex neurons produced by previous experience with amphetamine. J Neurosci 1997; 17: 8491–8497. 9334421 10.1523/JNEUROSCI.17-21-08491.1997 PMC6573726

[bib30] Muhammad A, Kolb B. Maternal separation altered behavior and neuronal spine density without influencing amphetamine sensitization. Behav Brain Res 2011; 223: 7–16. 21515311 10.1016/j.bbr.2011.04.015

[bib31] Muhammad A, Kolb B. Mild prenatal stress-modulated behavior and neuronal spine density without affecting amphetamine sensitization. Dev Neurosci 2011; 33: 85–98. 21576912 10.1159/000324744

[bib32] Gourley SL, Swanson AM, Koleske AJ. Corticosteroid-induced neural remodeling predicts behavioral vulnerability and resilience. J Neurosci 2013; 33: 3107–3112. 23407965 10.1523/JNEUROSCI.2138-12.2013 PMC3711631

[bib33] Schoenbaum G, Setlow B. Cocaine makes actions insensitive to outcomes but not extinction: implications for altered orbitofrontal-amygdalar function. Cereb Cortex 2005; 15: 1162–1169. 15563719 10.1093/cercor/bhh216

[bib34] Zapata A, Minney VL, Shippenberg TS. Shift from goal-directed to habitual cocaine seeking after prolonged experience in rats. J Neurosci 2010; 30: 15457–15463. 21084602 10.1523/JNEUROSCI.4072-10.2010 PMC3073559

[bib35] Corbit LH, Chieng BC, Balleine BW. Effects of repeated cocaine exposure on habit learning and reversal by N-acetylcysteine. Neuropsychopharmacology 2014; 39: 1893–1901. 24531561 10.1038/npp.2014.37 PMC4059898

[bib36] Bourgeois JP, Goldman-Rakic PS, Rakic P. Synaptogenesis in the prefrontal cortex of rhesus monkeys. Cereb Cortex 1994; 4: 78–96. 8180493 10.1093/cercor/4.1.78

[bib37] Casey BJ, Giedd JN, Thomas KM. Structural and functional brain development and its relation to cognitive and development. Biol Psychol 2000; 54: 241–257. 11035225 10.1016/s0301-0511(00)00058-2

[bib38] Giedd JN. Structural magnetic resonance imaging of the adolescent brain. Ann N Y Acad Sci 2004; 4: 78–96. 10.1196/annals.1308.009 15251877

[bib39] van Eden CG, Uylings HBM. Postnatal volumetric development of the prefrontal cortex in the rat. J Comp Neurol 1985; 241: 268–274. 4086657 10.1002/cne.902410303

[bib40] Paus T, Keshavan M, Giedd JN. Why do many psychiatric disorders emerge during adolescence? Nat Rev Neurosci 2008; 9: 947–957. 19002191 10.1038/nrn2513 PMC2762785

[bib41] Gourley SL, Olevska A, Warren MS, Taylor JR, Koleske AJ. Arg kinase regulates prefrontal dendritic spine refinement and cocaine-induced plasticity. J Neurosci 2012; 32: 2315–2323. 10.1523/JNEUROSCI.2730-11.2012 PMC3386297 22396406

[bib42] Gourley SL, Taylor JR. Going and stopping: dichotomories in behavioral control by the prefrontal cortex. Nat Neurosci 2016; 19: 656–664. 29162973 10.1038/nn.4275 PMC5087107

[bib43] CASACASA analysis of the National Survey on Drug Use and Health (NSDUH), 2009. U.S. Department of Health and Human Services, Substance Abuse and Mental Health Services Administration: Rockville, MD, USA, 2011.

[bib44] DePoy LM, Gourley SL. Cytoskeletal plasticity in the prefrontal cortex is associated with psychostimulant exposure. Traffic 2015; 16: 919–940. 25951902 10.1111/tra.12295 PMC4849269

[bib45] Crombag HS, Gorny G, Li Y, Kolb B, Robinson TE. Opposite effects of amphetamine self-administration experience on dendritic spines in the medial and orbital prefrontal cortex. Cereb Cortex 2005; 15: 341–348. 15269111 10.1093/cercor/bhh136

[bib46] Gourley SL, Koleske AJ, Taylor JR. Loss of dendrite stabilization by the Abl-related gene (Arg) kinase regulates behavioral flexibility and sensitivity to cocaine. Proc Natl Acad Sci USA 2009; 106: 16859–16864. 19805386 10.1073/pnas.0902286106 PMC2742404

[bib47] Bhatt DH, Zhang S, Gan WB. Dendritic spine dynamics. Ann Rev Physiol 2009; 77: 261–282. 10.1146/annurev.physiol.010908.163140 19575680

[bib48] Gremel CM, Costa RM. Orbitofrontal and striatal circuits dynamically encode the shift between goal-directed and habitual actions. Nat Commun 2013; 4: 2264. 23921250 10.1038/ncomms3264 PMC4026062

[bib49] Winstanley CA, LaPlant Q, Theobald DE, Green TA, Bachtell RK, Perrotti LI et al. ΔFosB induction in orbitofrontal cortex mediates tolerance to cocaine-induced cognitive dysfunction. J Neurosci 2007; 27: 10497–10507. 17898221 10.1523/JNEUROSCI.2566-07.2007 PMC6673166

[bib50] DePoy LM, Perszyk RE, Zimmermann KS, Koleske AJ, Gourley SL. Adolescent cocaine exposure simplifies orbitofrontal cortical dendritic arbors. Front Pharmacol 2014; 5: 228. 25452728 10.3389/fphar.2014.00228 PMC4233985

[bib51] Shors TJ, Chua C, Falduto J. Sex differences and opposite effects of stress on dendritic spine density in the male versus female hippocampus. J Neurosci 2001; 21: 6292–6297. 11487652 10.1523/JNEUROSCI.21-16-06292.2001 PMC6763131

[bib52] Munoz-Cueto JA, Garcia-Segura LM, Ruiz-Marcos A. Regional sex differences in spine density along the apical shaft of visual cortex pyramids during postnatal development. Brain Res 1991; 540: 41–47. 2054631 10.1016/0006-8993(91)90490-m

[bib53] Markham JA, Mullins SE, Koenig JI. Periadolescent maturation of the prefrontal is sex-specific and is disrupted by prenatal stress. J Comp Neurol 2013; 521: 1828–1843. 23172080 10.1002/cne.23262 PMC4479145

[bib54] Mychasiuk R, Muhammad A, Carroll C, Kolb B. Does prenatal nicotine exposure alter the brain's response to nicotine in adolescence? A neuroanatomical analysis. Eur J Neurosci 2013; 38: 2491–2503. 23679187 10.1111/ejn.12245

[bib55] Gabbott PL, Warner TA, Busby SJ. Amygdala input monosynaptically innervates parvalbumin immunoreactive local circuit neurons in rat medial prefrontal cortex. Neuroscience 2006; 139: 1039–1048. 16527423 10.1016/j.neuroscience.2006.01.026

[bib56] Mashhoon Y, Wells AM, Kantak KM. Interaction of the rostral basolateral amygdala and prelimbic prefrontal cortex in regulating reinstatement of cocaine-seeking behavior. Pharmacol Biochem Behav 2010; 96: 347–353. 20600250 10.1016/j.pbb.2010.06.005 PMC2940107

[bib57] Stefanik MT, Kalivas PW. Optogenetic dissection of basolateral amygdala projections during cue-induced reinstatement of cocaine seeking. Front Behav Neurosci 2013; 7: 213. 24399945 10.3389/fnbeh.2013.00213 PMC3871970

[bib58] Martin-Garcia E, Courtin J, Renault P, Fiancette JF, Wurtz H, Simonnet A et al. Frequency of cocaine self-administration influences drug seeking in the rat: optogenetic evidence for a role of the prelimbic cortex. Neuropsychopharmacology 2014; 39: 2317–2330. 24633559 10.1038/npp.2014.66 PMC4138740

[bib59] Robinson TE, Kolb B. Alterations in the morphology of dendrites and dendritic spines in the nucleus accumbens and prefrontal cortex following repeated treatment with amphetamine or cocaine. Eur J Neurosci 1999; 11: 1598–1604. 10215912 10.1046/j.1460-9568.1999.00576.x

[bib60] Li Y, Kolb B, Robinson TE. The location of persistent amphetamine-induced changes in the density of dendritic spines on medium spiny neurons in the nucleus accumbens and caudate-putamen. Neuropsychopharmacology 2003; 28: 1082–1085. 12700699 10.1038/sj.npp.1300115

[bib61] Kolb B, Gorny G, Li Y, Samaha AN, Robinson TE. Amphetamine or cocaine limits the ability of later experience to promote structural plasticity in the neocortex and nucleus accumbens. Proc Natl Acad Sci USA 2003; 100: 10523–10528. 12939407 10.1073/pnas.1834271100 PMC193594

[bib62] Liu RJ, Aghajanian GK. Stress blunts serotonin- and hypocretin-evoked EPSCs in prefrontal cortex: role of corticosterone-mediated apical dendritic atrophy. Proc Natl Acad Sci USA 2008; 105: 359–364. 18172209 10.1073/pnas.0706679105 PMC2224217

[bib63] Quinn JJ, Hitchcott PK, Umeda EA, Arnold AP, Taylor JR. Sex chromosome complement regulates habit formation. Nat Neurosci 2007; 10: 1398–1400. 17952068 10.1038/nn1994

[bib64] Holtz NA, Carroll ME. Escalation of i.v. cocaine intake in per-adolescent vs. adult rats selectively bred for high (HiS) vs. low (LoS) saccharin intake. Psychopharmacology 2013; 227: 243–250. 23307070 10.1007/s00213-012-2958-8 PMC4583775

[bib65] Perry JL, Morgan AD, Anker JJ, Dess NK, Carroll ME. Escalation of i.v. cocaine self-administration and reinstatement of cocaine-seeking behavior in rats bred for high and low saccharin intake. Psychopharmacology 2006; 186: 235–245. 16596398 10.1007/s00213-006-0371-x

[bib66] Holtz NA, Carroll ME. Baclofen has opposite effects on escalation of cocaine self-administration: increased intake in rats selectively bred for high (HiS) saccharin intake and decreased intake in those selected for low (LoS) saccharin intake. Pharmacol Biochem Behav 2011; 100: 275–283. 21924281 10.1016/j.pbb.2011.08.028 PMC3206724

[bib67] Pitts EG, Taylor JR, Gourley SL. Prefrontal cortical BDNF: a regulatory key in cocaine- and food-reinforced behaviors. Neurobiol Dis 2016; 91: 326–335. 26923993 10.1016/j.nbd.2016.02.021 PMC4913044

[bib68] Li X, Wolf ME. Multiple faces of BDNF in cocaine addiction. Behav Brain Res 2015; 279: 240–254. 25449839 10.1016/j.bbr.2014.11.018 PMC4277902

[bib69] Zimmermann KS, Yamin JA, Rainnie DG, Ressler KJ, Gourley SL. Connections of the mouse orbitofrontal cortex and regulation of goal-directed action selection by BDNF-TrkB. Biol Psychiatry; e-pub ahead of print 18 November 2015; doi: 10.1016/j.biopsych.2015.10.026. 10.1016/j.biopsych.2015.10.026 PMC4871791 26786312

[bib70] Otis JM, Fitzgerald MK, Mueller D. Infralimbic BDNF/TrkB enhancement of GluN2B currents facilitates extinction of a cocaine-conditioned place preference. J Neurosci 2014; 34: 6057–6064. 24760865 10.1523/JNEUROSCI.4980-13.2014 PMC3996223

