# Supplementary material for: Adolescent cocaine self-administration induces habit behavior in adulthood: sex differences and structural consequences
Source: Transl Psychiatry. 2016 Aug 30;6(8):e875–. doi: 10.1038/tp.2016.150 (PMC5022090; doi:10.1038/tp.2016.150)
Supplement: Supplementary Figure 1 [file tp2016150x1.doc]

| **Figure** | **Sex** | **Cocaine Concentration(s)** | **Cocaine Exposure Method** | **Age of Cocaine** | **Start of**  **Subsequent Testing in Adulthood** | **Post-Euthan-asia** |
| --- | --- | --- | --- | --- | --- | --- |
| **1a,b** | female | sucrose (*n*=21) or 7.5 µg/ml cocaine (*n*=16) or 75 µg/ml cocaine (*n*=16) | oral cocaine self-administration | P31-42 | n/a |  |
| **1c-i** | male | sucrose (*n*=8) or 7.5 µg/ml cocaine (*n*=7 stable, 8 escalating) | oral cocaine self-administration | P31-42 | P56, food-reinforced instrumental conditioning |  |
| **2a-i** | female | sucrose (total *n*=21) or 7.5 µg/ml cocaine (*n*=8 stable, 8 escalating) or 75 µg/ml cocaine (*n*=8 stable, 8 escalating) | oral cocaine self-administration | P31-42 | P56, food-reinforced instrumental conditioning |  |
| **2j,k** | female | sucrose or 7.5 µg/ml cocaine (*n*=4/group) | *i.p.* injections matched to oral cocaine doses | P31-42 | P56, food-reinforced instrumental conditioning |  |
| **3a-c** | female | sucrose or 7.5 µg/ml cocaine (*n*=5/group) | oral cocaine self-administration, then *i.p.* injections | P31-42 | P56, cocaine-induced locomotor sensitization and cocaine-conditioned object preference |  |
| **3d,e** | female | sucrose (*n*=10) or 7.5 µg/ml cocaine (*n*=6 stable, 4 escalating; groups are uneven due to unexpected deaths) | oral cocaine self-administration | P31-42 | P56, food-reinforced instrumental conditioning, then context-induced reinstatement |  |
| **4a-c** | female | sucrose (*n*=8) or 7.5 µg/ml cocaine (*n*=7 stable following unexpected death of one animal, 9 escalating). Total spine counts are provided in the main text. | oral cocaine self-administration | P31-42 | P56, food-reinforced instrumental conditioning | At P75, image dendritic spines |
| **4d** | male | sucrose or 7.5 µg/ml cocaine (*n*=4/group). Total spine counts are provided in the main text. | oral cocaine self-administration | P31-42 | P56, food-reinforced instrumental conditioning | At P75, image dendritic spines |
| **5** | female | sucrose or 7.5 µg/ml cocaine (*n*=9/group, or 8 in the case of non-real numbers resulting from normalization) | oral cocaine self-administration | P31-42 | P56, food-reinforced instrumental conditioning, then context-induced reinstatement |  |
| **Suppl. fig.2** | female | sucrose (*n*=6) or 7.5 µg/ml cocaine (*n*=7 stable, 6 escalating) | oral cocaine self-administration | P42-53 | P67, food-reinforced instrumental conditioning, then context-induced reinstatement |  |
| **Suppl. fig.3** | female | Sensitization curves associated with fig.3a-c | |  |  |  |

**Suppl. Fig.1. A summary of experiments in DePoy *et al.***
