# Supplementary material for: Adolescent cocaine self-administration induces habit behavior in adulthood: sex differences and structural consequences
Source: Transl Psychiatry. 2016 Aug 30;6(8):e875–. doi: 10.1038/tp.2016.150 (PMC5022090; doi:10.1038/tp.2016.150)
Supplement: Supplementary Figure 2 [file tp2016150x2.doc]

**Suppl. Fig.2. Periadolescent mice will respond for an oral cocaine reinforcer.**

**DePoy et al.**

**
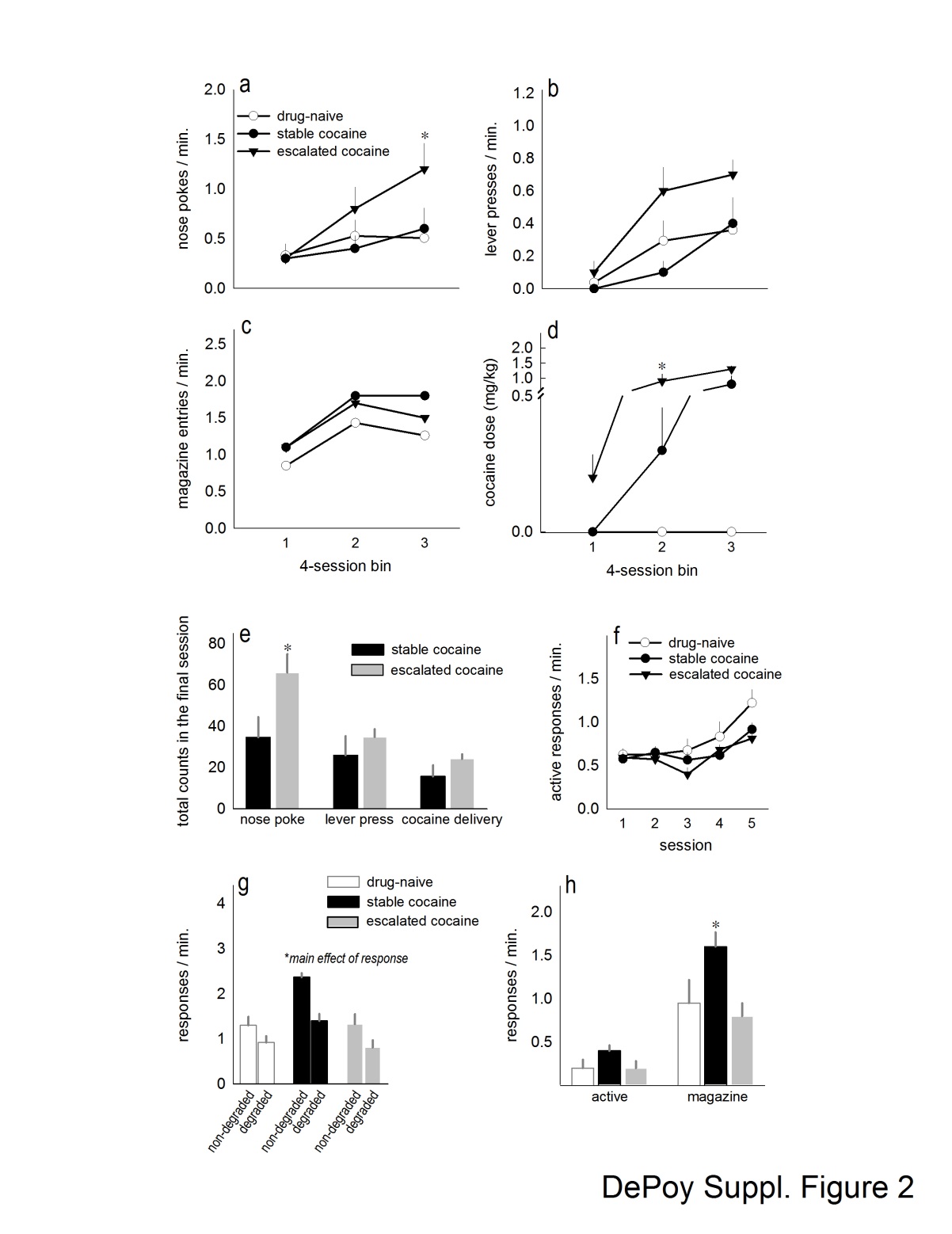
**

In a final series of experiments, we trained a group of older female *periadolescent* (P42; per main text ref. 17) mice to self-administer 7.5 μg/ml cocaine. (a) Notably, responding for cocaine was more homogenous: For example, after a median split based on total nose-poke responses, nose-poking profiles diverged as expected [interaction F_(2,16)_=4.4,p=0.04], but (b) we identified only a strong trend for an effect of group on lever pressing [main effect F_(2,16)_=3.3,p=0.06] and (c) no effect on magazine head entries [Fs<1]. (d) The dose of cocaine acquired rose more rapidly in escalating mice than stable mice, but unlike in adolescent populations, “stable” periadolescent mice ultimately acquired equal amounts of cocaine per session by the end of the self-administration period [interaction F_(1,11)_=6.4,p=0.03]. (e) Indeed, on the last training day, only total nose pokes differed between cocaine-reinforced groups (unpaired t-test, p=0.04), while total lever presses and total cocaine deliveries did not differ (ps>0.2).

(f) Two weeks later, mice were tested for sensitivity to response-outcome contingency degradation. There were no differences in instrumental response acquisition (F<1), and (g) mice were subsequently sensitive to response-outcome contingency degradation, regardless of cocaine status [main effect of response F_(1,15)_=18.9,p<0.001; interaction p=0.3]. These findings parallel epidemiological evidence that individuals that initiate cocaine use as young adults are at lower risk of transitioning from occasional to routine cocaine use, relative to individuals that initiate use in adolescence (main text 8). (h) One notable caveat, however, is that like younger cohorts, stable responders showed evidence of increased reward-seeking behavior when returned to the cocaine-associated chambers; specifically, they generated more magazine head entries than other groups [main effect F_(2,16)_=4.3,p=0.03]. Nose-poking did not differ between groups [main effect F_(2,16)_=1.5,p=0.3].
