# Supplementary material for: Adolescent cocaine self-administration induces habit behavior in adulthood: sex differences and structural consequences
Source: Transl Psychiatry. 2016 Aug 30;6(8):e875–. doi: 10.1038/tp.2016.150 (PMC5022090; doi:10.1038/tp.2016.150)
Supplement: Supplementary Figure 3 [file tp2016150x3.doc]

**Suppl. Fig.3. Locomotor sensitization curves associated with main text figure 3.**

**DePoy et al.**

**
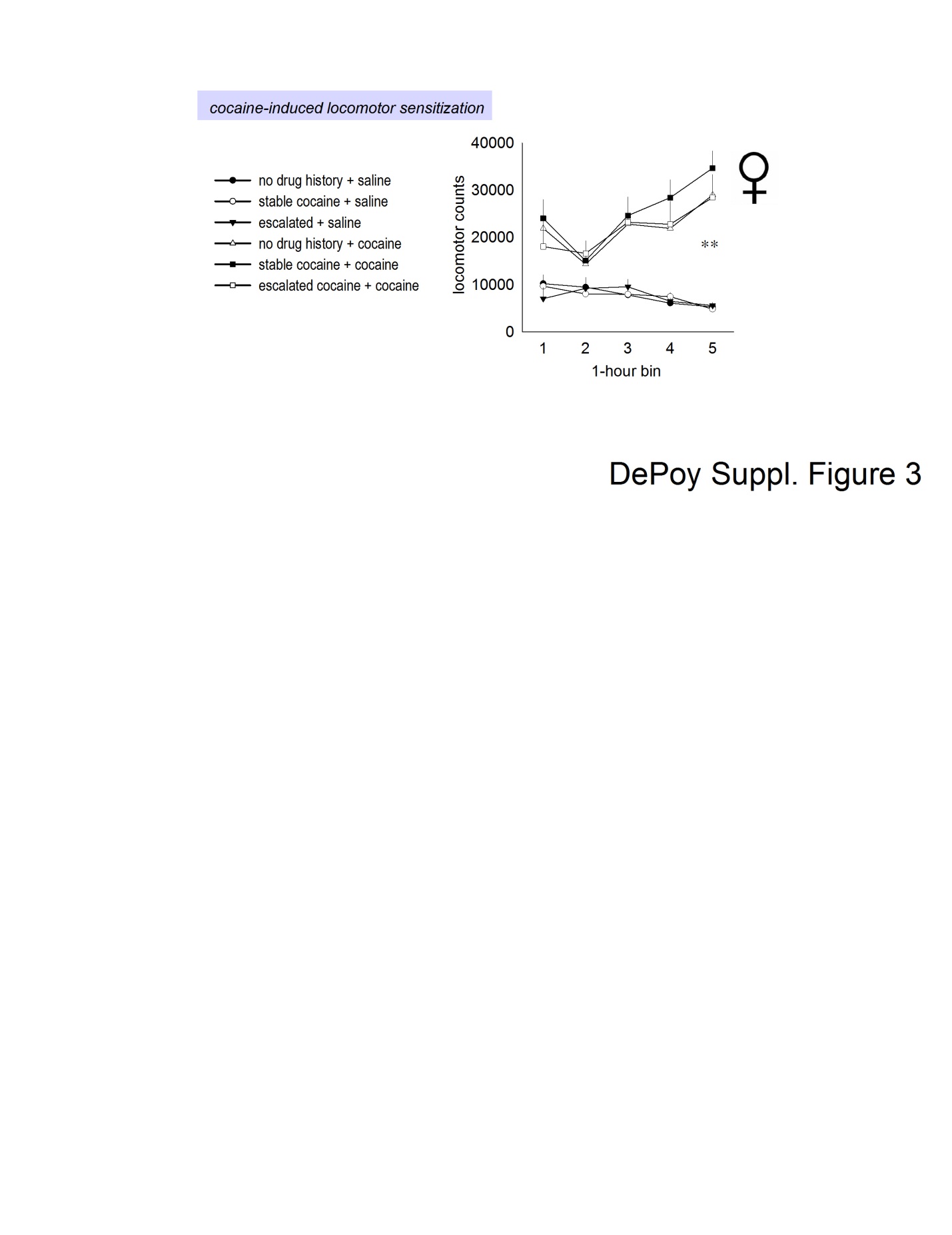
**Mice were administered saline and cocaine daily, separated by >3 hours, for 5 sequential days, then placed in locomotor monitoring chambers. Locomotor counts progressively increased across days, particularly following cocaine. We detected no group differences. **p<0.001 main effect of day.
